# Impact of Ball-Milling and Thermal Hydrolysis on Physicochemical Properties and Anaerobic Digestion Kinetics of Mixed Slaughterhouse and Agricultural Wastes

**DOI:** 10.3390/bioengineering13030326

**Published:** 2026-03-11

**Authors:** Sang Heon Lee, Oh Hyun Gweon, Hye Sun Lee, Byoung Seung Jeon, Youngwook Go, Chang Sook Jin, Youngseob Yu, Byoung-In Sang, Jin Hyung Lee

**Affiliations:** 1 Korea Institute of Ceramic Engineering and Technology, Cheongju 28160, Republic of Korea; dltkggjs4709@kicet.re.kr (S.H.L.); ohhyunggweon@kicet.re.kr (O.H.G.); hslee@kicet.re.kr (H.S.L.); a1trust@kicet.re.kr (B.S.J.); 2 Department of Chemical Engineering, Hanyang University, Seoul 04763, Republic of Korea; youngwook@hanyang.ac.kr (Y.G.); biosang@hanyang.ac.kr (B.-I.S.); 3 KEC System Co. Ltd., Hwasung 18244, Republic of Korea; csjinfj@hanmail.net (C.S.J.); u0seob@naver.com (Y.Y.)

**Keywords:** anaerobic co-digestion, waste valorization, ball-milling, thermal hydrolysis, slaughterhouse waste, agricultural residues, reaction kinetics

## Abstract

Slaughterhouse by-products are promising feedstocks for anaerobic digestion due to their high lipid and protein content. However, their complex structures often limit hydrolysis, and excessive pretreatment can induce inhibitory conditions. This study evaluates the effects of ball-milling (BM), ball-milling with water (BM + water), and combined thermal hydrolysis and ball-milling (THP + BM) on the digestion performance of a mixed substrate of slaughterhouse and agricultural wastes. The results demonstrate that all BM-based pretreatments significantly improved digestion kinetics, reducing the lag phase by 26–66% and shortening the *T*_50_ values by approximately 40% compared to the untreated substrate. While no statistically significant differences were observed in the ultimate methane yield, the onset of methanogenesis was markedly accelerated in the BM and BM + water treatments. In contrast, despite achieving superior solubilization, the THP + BM treatment failed to provide proportional kinetic enhancements. This was attributed to a severe initial metabolic imbalance—characterized by a pH drop below the inhibitory threshold (6.33)—which induced physiological stress and delayed the functional recovery of methanogens. These findings indicate that while ball-milling effectively facilitates digestion initiation by enhancing physical accessibility, the intensity of combined thermal-mechanical processes must be strategically optimized. For high-strength organic biomass, managing pretreatment severity is crucial to prevent initial acid stress and maximize process efficiency.

## 1. Introduction

As climate change intensifies worldwide, international efforts to achieve carbon neutrality have expanded significantly. According to the Intergovernmental Panel on Climate Change (IPCC) Sixth Assessment Report (AR6), the energy sector originates approximately 34% of global greenhouse gas emissions, while the waste sector accounts for about 3% [1,2]. Methane emitted during waste management is classified as a short-lived climate forcer (SLCF) and has a global warming potential nearly 80 times greater than that of CO_2_ over a 20-year horizon, making it a critical target for short-term climate mitigation strategies [3]. In this context, anaerobic digestion (AD) has attracted increasing attention as a renewable energy technology that can simultaneously reduce methane emissions from organic waste and recover energy in the form of biogas. AD stabilizes organic waste and prevents unintended methane emissions associated with landfilling. In addition, it enables controlled biogas recovery, contributing to climate change mitigation and sustainable waste management [4,5,6,7].

To enhance carbon neutrality through waste-to-energy pathways, identifying and utilizing diverse unutilized biomass sources is imperative. According to the Food and Agriculture Organization Corporate Statistical Database (FAOSTAT), the global generation of slaughterhouse by-products (hides, skins, and fats) has steadily increased with the expansion of livestock production [8]. These by-products are characterized by high protein and lipid contents, rendering them organic feedstocks with high theoretical methane potential [9,10]. Nevertheless, in practical waste management, slaughterhouse by-products are still predominantly treated through low-value disposal methods such as landfilling and incineration. Such practices fail to recover the inherent energy potential of these materials while contributing to greenhouse gas emissions and additional environmental burdens. While these by-products serve as excellent feedstocks for energy production via AD due to their high nutrient content, several operational challenges associated with the AD process have been reported. For instance, long-chain fatty acids (LCFAs) generated during lipid hydrolysis can adsorb onto microbial cell membranes or exhibit surfactant-like properties, thereby inhibiting methanogenic activity [11]. In addition, excessive accumulation of ammonia released during protein degradation may impair the overall stability of the AD process [12,13]. Accordingly, despite their high theoretical methane potential, slaughterhouse by-products are recognized as substrates with a high risk of process inhibition in practical AD applications.

To overcome these limitations, co-digestion strategies involving slaughterhouse by-products and other organic substrates—specifically unutilized agricultural residues—have been proposed to balance nutrient composition and mitigate inhibitory effects [9,14]. While co-digestion adjusts the C/N ratio, several studies have also employed thermal pretreatment to improve substrate degradability [15,16]. However, the diverse physical and chemical properties of these mixed substrates often lead to structural recalcitrance and physical heterogeneity, which cannot be fully addressed by thermal methods alone. Ball milling (BM) has been reported as an effective physical pretreatment for improving the degradation of various organic wastes by reducing particle size and enhancing homogeneity [16,17,18]. Yet, its application to complex protein-lipid matrices, such as those found in slaughterhouse-based mixed substrates, remains poorly understood compared to its use in lignocellulosic biomass. Physical heterogeneity in such mixed substrates may induce imbalances during the initial hydrolysis and acidogenesis stages, potentially delaying methane production. From this perspective, BM can be considered a strategic physical pretreatment capable of restructuring the complex matrix, thereby improving substrate–microorganism contact and mass transfer during the early stages of digestion.

Therefore, this study aimed to investigate the synergistic integration of mechanical BM and thermal hydrolysis on a mixed substrate composed of slaughterhouse by-products and agricultural residues. By analyzing changes in particle size distribution and substrate homogeneity, and assessing the impacts on AD kinetics through BMP tests, this research identifies the optimal pretreatment intensity required to maximize energy recovery. Through this approach, the role of physical-thermal synergy in the efficient conversion of unutilized biomass blends was systematically investigated.

## 2. Materials and Methods

### 2.1. Substrates and Inoculum

Agricultural residues and five types of slaughterhouse waste—including stomach contents, liver, lung, residual cake, and skin waste—were used as feedstocks. Agricultural residues, consisting of vegetable and fruit wastes, were collected from Goyang Agricultural Market (Goyang, Republic of Korea). Slaughterhouse waste was obtained from large-scale facilities in Anseong and Incheon, Republic of Korea, while skin waste was provided by Sammi Industry (Ansan, Republic of Korea). To characterize the physicochemical properties of each biomass, total Kjeldahl nitrogen (TKN), moisture content, total solids (TSs), volatile solids (VSs), the VS/TS ratio, and the TKN/VS ratio were determined. To ensure the practical relevance and scalability of the results, the mixing ratio of the substrates was determined based on the design specifications of an 80 ton/day industrial-scale anaerobic digestion facility currently under development. The proportions were calculated considering the actual daily supply capacities officially committed by the respective feedstock providers to reflect realistic waste streams. Consequently, the final mixed substrate was composed of agricultural residue (46 wt%) and five types of slaughterhouse by-products: stomach contents (21 wt%), residual cake (13 wt%), liver (8 wt%), lung (4 wt%), and skin waste (4 wt%).

The inoculum consisted of anaerobic sewage sludge collected from the Jungrang Wastewater Treatment Plant (Seoul, Republic of Korea). To stabilize methanogenic activity, the sludge was pre-incubated at 37 °C for 4 days while being continuously flushed with N_2_ gas to maintain strictly anaerobic conditions prior to use. The inoculum exhibited a stable initial pH and robust buffering capacity, ensuring a suitable microbial environment for the digestion tests.

### 2.2. Pretreatment Method

To investigate the effects of different pretreatment strategies on anaerobic digestion, three distinct methods were employed: (1) ball milling (BM), (2) ball milling with water addition (BM + water), and (3) combined thermal hydrolysis and ball milling (THP + BM). All pretreatment processes were applied to the mixed substrate prepared as described in Section 2.1.

#### 2.2.1. Ball Milling (BM and BM + Water)

Ball milling was performed using an attrition mill (KMD-10S, Korea Powder Machinery Co., Ltd., Incheon, Republic of Korea) equipped with iron balls (15 mm in diameter). For the BM condition, the raw mixed substrate was directly milled. For the BM + water condition, 20% (*w*/*w*) distilled water was added to the substrate prior to milling to evaluate the effect of moisture content on grinding efficiency. In both cases, the volume ratio of substrate to iron balls was maintained at 6:4. This ratio was specifically selected to evaluate a higher substrate throughput compared to conventional laboratory ratios (e.g., 1:1), aiming to better simulate the operational conditions of a high-capacity industrial attrition mill. The milling was conducted at a rotational speed of 300 rpm for 10 min.

#### 2.2.2. Combined Pretreatment (THP + BM)

For the integrated pretreatment (THP + BM), thermal hydrolysis was performed as a first step. Distilled water (20% *w*/*w*) was added to the mixed substrate, and the mixture was treated in a high-pressure reactor (R-201, ChemRe System Inc., Anyang, Republic of Korea) at 160 °C for 15 min. After the reactor cooled to room temperature, the thermally hydrolyzed substrate was subjected to ball milling under the same conditions (300 rpm, 10 min) to assess the synergistic effect of thermal and mechanical energy.

### 2.3. Analytical Methods and Experimental Design

The pretreated substrates were characterized by analyzing total solids (TSs), volatile solids (VSs), and particle size. TSs and VSs were determined according to the standard methods described by the American Public Health Association (APHA) [19]. Particle size distribution was measured using a laser diffraction analyzer (LA-960, Horiba Ltd., Kyoto, Japan). To evaluate the solubilization of organic matter induced by the pretreatments, the solubilized fractions were separated from the mixed substrate via vacuum filtration (Whatman No. 41, 20 µm). The resulting filtrates (liquid phase) were subsequently analyzed for pH, soluble total organic carbon (S-TOC), soluble carbohydrates, and soluble proteins. S-TOC was determined using a TOC analyzer (TOC-L, Shimadzu Co. Ltd., Kyoto, Japan), and pH was measured with a pH meter (F-71, Horiba Ltd., Kyoto, Japan). Soluble carbohydrates were quantified using a modified phenol-sulfuric acid method [20] with D-(+)-glucose (Sigma-Aldrich Co. LLC, St Louis, MA, USA) as the standard. Soluble protein concentrations were determined using a bicinchoninic acid (BCA) protein assay kit (Pierce™, Thermo Fisher Scientific) following the manufacturer’s protocol. Absorbance for both assays was measured using a microplate spectrophotometer (Multiskan SkyHigh, Thermo Fisher Scientific, Waltham, MA, USA).

Methane production performance was evaluated for four experimental conditions: raw substrate, BM, BM + water, and THP + BM. Biochemical methane potential (BMP) tests were conducted in 200 mL serum bottles, each loaded with 80 mL of inoculum and 25 g of substrate. This resulted in a substrate-to-inoculum (S/I) ratio of 1.5–1.6 (VS basis), which was intentionally selected to enhance the sensitivity of the assay in detecting differences between pretreatment methods. All experiments were performed in triplicate. After flushing the headspace with N_2_ gas, the bottles were sealed and incubated at 37 °C in a shaking incubator (SI-300, JeioTech Co. Ltd., Daejeon, Republic of Korea) at 70 rpm. Biogas volume was measured periodically using a syringe, and the gas composition (CH_4_, CO_2_, and N_2_) was analyzed using gas chromatography with a thermal conductivity detector (GC-TCD; iGC-7200A, DS Science Co. Ltd., Seoul, Republic of Korea).

To monitor the stability of the AD process, temporal variations in pH and volatile fatty acids (VFAs) in the digestate were quantitatively analyzed to elucidate key factors influencing methane production under different pretreatment conditions. VFAs were quantified using a gas chromatograph with a flame ionization detector (GC-FID; GC-7810, Agilent Technologies, Santa Clara, CA, USA) equipped with a DB-FATWAX Ultra Inert column.

### 2.4. Calculation Method

The modified Gompertz model is a nonlinear function commonly used to describe the temporal evolution of methane production based on the lag phase and the exponential growth phase [21]. In this study, the modified Gompertz model was applied to fit the cumulative methane production curves of each sample.

(1) $$\begin{array}{c} M\left(t\right)={\;P}_{m}\;\cdot \;exp\left\{-\exp\left[\frac{R_{m}\;\cdot \;e}{P_{m}}\left(\lambda -t\right)+1\right]\right\} \end{array}$$

where *M(t)* is the cumulative methane production at digestion time *t* (mL g^−1^VS), *P_m_* is the maximum methane production potential (mL g^−1^VS), *R_m_* is the maximum methane production rate (mL g^−1^VS·d^−1^), *e* is Euler’s number (≈2.7183), λ is the lag phase duration (d), and *t* is the digestion time (d). The kinetic parameters (*P_m_*, *R_m_*, and λ) were estimated for each substrate through non-linear regression analysis. The goodness of fit was evaluated using the coefficient of determination (*R*^2^). In this study, this model was specifically selected over first-order kinetic models because it effectively accounts for the distinctive lag phase observed in our experimental curves, which is a critical parameter for evaluating the digestibility of complex mixed substrates.

The particle size distribution and characteristic methane production kinetics were evaluated using D-values and T-values, respectively. D-values (D_10_, D_50_, and D_90_) represent the particle diameters at which 10%, 50%, and 90% of the cumulative volume of the sample is reached. For instance, D_50_ denotes the median particle size, while D_10_ and D_90_ indicate the sizes below which 10% and 90% of the particles are distributed, providing a measure of the physical refinement achieved by pretreatment. T-values (T_50_ and T_80_) were defined as the time (d) required to reach 50% and 80% of the maximum cumulative methane production (*P_m_*), respectively. These values serve as indicators of the methane production rate and the efficiency of the anaerobic digestion process. Both D-values and T-values were determined using linear interpolation between the adjacent data points surrounding the target cumulative thresholds. For interpolation, data points satisfying X_0_ < X < X_1_ were used, where Y_0_ and Y_1_ correspond to the measured values at X_0_ and X_1_, respectively.

(2) $$Y=Y_{0\;}+\frac{\left(X-X_{0}\right)}{\left(X_{1\;}-\;X_{0}\right)}\times (Y_{1}-Y_{0})$$

where X_0_ and X_1_ are the cumulative fractions just below and above the target, and Y_0_ and Y_1_ are the corresponding measured values.

To evaluate the changes in particle size uniformity induced by each pretreatment method, the SPAN value was calculated using Equation (3):

(3) $$\mathrm{SPAN}\;=\;\frac{{(D}_{90}-D_{10})}{D_{50}}$$

where the D-values corresponding to the cumulative diameters defined previously. A lower SPAN value indicates a more uniform particle size distribution, representing a higher precision in the mechanical or thermal-mechanical disintegration of the substrate.

### 2.5. Statistical Method

Statistical analysis were performed to evaluate the significant differences among the pretreatment conditions. All statistical tests were conducted using Jamovi software (version 2.5.6). Prior to parametric analysis, the data were examined to verify the fundamental assumptions required for the analysis of variance (ANOVA). Normality was assessed using the Shapiro–Wilk test, while the homogeneity of variances was evaluated using the Leven’s test and Bartlett’s test. When both assumptions of normality and homogeneity of variances were satisfied, one-way ANOVA was conducted, followed by Tukey’s honestly significant difference (HSD) test for post hoc multiple comparisons. For datasets that violated the assumption of homogeneity of variances, Welch’s ANOVA was applied, and pairwise comparisons were subsequently performed using the Games-Howell post hoc test. For all tests, statistical significance was determined at *p* < 0.05.

## 3. Results and Discussion

### 3.1. Physicochemical Characteristics of Raw and Pretreated Substrates

#### 3.1.1. Properties of Slaughterhouse By-Products and Agricultural Residues

The physicochemical characteristics of the slaughterhouse by-products and agricultural residues showed distinct differences (Table 1). Overall, the slaughterhouse by-products were characterized by higher total solids (TSs) and volatile solids (VSs) contents compared to the agricultural residues. Specifically, the residual cake exhibited the highest TS and VS concentrations, reaching 43.3% and 43.1% (*w*/*w*), respectively.

A notable feature of the slaughterhouse by-products, excluding the stomach contents, was their high nitrogen content. The liver, skin waste, and lung samples showed remarkably high total Kjeldahl nitrogen (TKN) concentrations of 29,632, 27,624, and 23,782 mg/kg, respectively. Consequently, their TKN/VS ratios exceeded 10%, indicating a high potential for ammonia inhibition if digested alone. In contrast, the stomach contents showed a relatively low TKN concentration (4508 mg/kg), and the agricultural residues were characterized by a high moisture content (90.6%) with no detectable TKN.

While the high organic content of slaughterhouse by-products makes them favorable substrates for methane production, their elevated TKN levels often act as a limiting factor in anaerobic digestion. Therefore, in this study, agricultural residues were utilized as a nitrogen-free co-substrate to balance the nutrient composition and mitigate potential ammonia toxicity, thereby optimizing the overall substrate characteristics for stable methane production.

#### 3.1.2. Physical Disintegration: Particle Size Distribution and Uniformity

**Figure 1:**
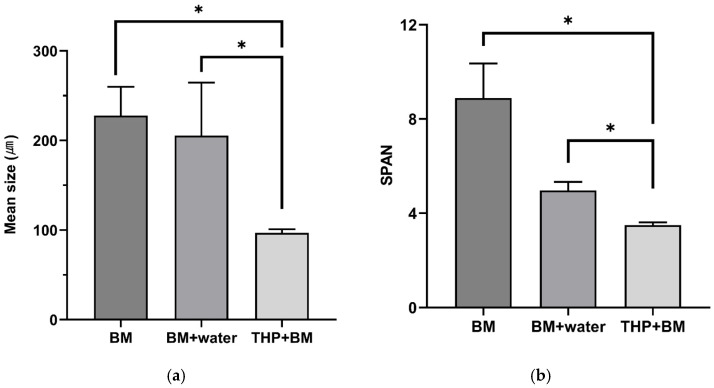
Comparison of mean particle size and SPAN value of the mixed substrate according to different pretreatment methods. (**a**,**b**) indicate mean particle size and SPAN value, respectively. Error bars represent the standard deviation of triplicate measurement (*: *p* > 0.05).

The physical structure of the mixed substrate, specifically particle size and homogeneity, was significantly affected by the different pretreatment methods applied (Figure 1). As the pretreatment shifted from simple mechanical milling (BM) to the combined thermal and mechanical approach (THP + BM), a progressive reduction in particle size was observed. The THP + BM treatment achieved the smallest mean particle size of 97.0 μm, which was substantially lower than those of BM (227.8 μm) and BM + water (205.4 μm).

A similar trend was observed for the SPAN value, which represents the uniformity of the particle size distribution. As detailed in Table S1, the individual cumulative diameters (D_10_, D_50_, and D_90_) showed a consistent decrease as the pretreatment method shifted from BM to THP + BM. Consequently, the SPAN value decreased from 8.89 (BM) and 4.97 (BM + water) to 3.51 after the combined THP and BM treatment. A lower SPAN value signifies a narrower distribution, indicating that the combined thermal and mechanical pretreatment (THP + BM) resulted in a more uniformly disintegrated substrate. Such improved homogeneity is generally favorable for anaerobic digestion, as it ensures consistent physical conditions by minimizing substrate heterogeneity. Ultimately, this uniformity contributes to more stable and consistent biogas production throughout the digestion process [7].

The statistical significance of these physical changes was evaluated using ANOVA and post hoc tests. The differences between BM and BM + water were not statistically significant (*p* = 0.771 for mean size and *p* = 0.069 for SPAN value), whereas THP + BM showed a significant improvement compared to all other groups (*p* < 0.05). These results suggest that while simple ball milling (with or without water) has a limited impact on the substrate’s structure, the combined thermal and mechanical approach (THP + BM) is the most effective method specifically for physical disintegration. However, it is noteworthy that such intensive physical disruption and thermal exposure do not always translate proportionally to biological methane yield, a phenomenon that will be discussed further in the context of digestion performance (Section 3.2).

In contrast to these structural changes, no statistically significant alterations were observed in the organic content (TS and VS) of the substrate (Tables S2; *p* = 0.157 for TS and *p* = 0.060 for VS). This discrepancy between physical transformation and chemical stability indicates that the pretreatments selectively modified the physical structure—increasing the available surface area—without inducing any loss of organic matter. This preservation of the initial organic load is crucial, as it suggests that any subsequent changes in methane production are driven by improved biodegradability rather than a shift in the substrate’s organic concentration.

#### 3.1.3. Effects of Pretreatment on the Solubilization of Organic Fractions

**Figure 2:**
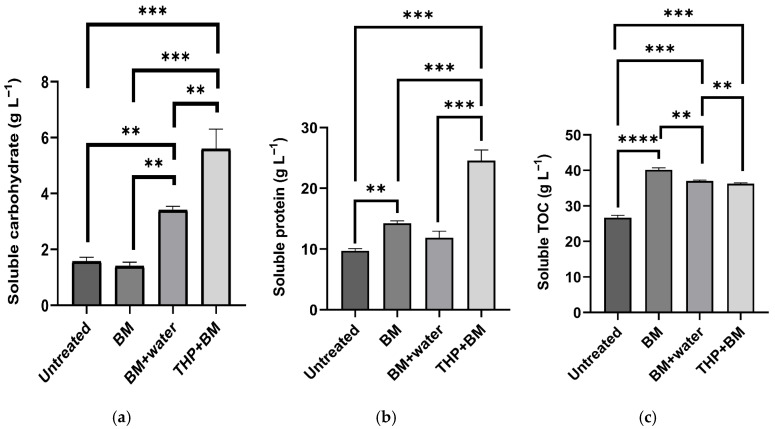
Concentration of solubilized organic fractions following different pretreatment methods: (**a**) soluble carbohydrates, (**b**): soluble proteins, and (**c**) soluble total organic carbon (S-TOC). Error bars represent the standard deviation of triplicate measurements (**: *p* < 0.05, ***: *p* < 0.01, ****: *p* < 0.0001).

The hydrolysis of complex organic matter into soluble forms is widely recognized as the rate-limiting step in the anaerobic digestion of solid-rich substrates. By transitioning particulate organic fractions into the liquid phase, pretreatments can bypass this slow enzymatic breakdown, thereby providing readily biodegradable substrates for acidogenic and methanogenic microorganisms [22]. In this study, significant changes in the concentrations of soluble organic compounds were observed following the pretreatments (Figure 2). The concentration of soluble carbohydrates increased in the order of untreated (1.6 g L^−1^) ≈ BM (1.4 g L^−1^) < BM + water (3.4 g L^−1^) < THP + BM (5.6 g L^−1^). Except for the comparison between BM and the untreated substrate (*p* = 0.947), statistically significant differences were observed among all other treatments (Figure 2a).

A similar trend was observed for soluble protein concentrations (Figure 2b). The highest concentration was achieved under the THP + BM treatment (24.6 g L^−1^), followed by BM (14.2 g L^−1^), BM + water (11.9 g L^−1^), and the untreated sample (9.7 g L^−1^). While no significant differences were found between the untreated and BM + water (*p* = 0.089) or between BM and BM + water (*p* = 0.063), all other comparisons showed statistically significant increases. These results indicate that the combination of thermal and mechanical energy in THP + BM was particularly effective in hydrolyzing macromolecular proteins and carbohydrates.

The soluble TOC (S-TOC) concentration also increased significantly in all pretreated samples compared to the untreated substrate (26.7 g L^−1^, *p* < 0.001). Interestingly, the highest S-TOC concentration was observed under the BM treatment (40.1 g L^−1^) (Figure 2c), whereas BM + water and THP + BM showed slightly lower values of 37.0 g L^−1^ and 36.3 g L^−1^, respectively. No statistically significant difference was observed between the BM + water and THP + BM treatments (*p* = 0.091).

Overall, the mechanical and hydrothermal pretreatments successfully enhanced the solubilization of organic matter, increasing its potential bioavailability. However, the varying distribution of S-TOC, proteins, and carbohydrates across treatments suggests that different pretreatment mechanisms induce distinct chemical transformations, which may lead to varying responses during the subsequent methane production phase.

### 3.2. Cumulative Methane Yield and Potential

**Figure 3:**
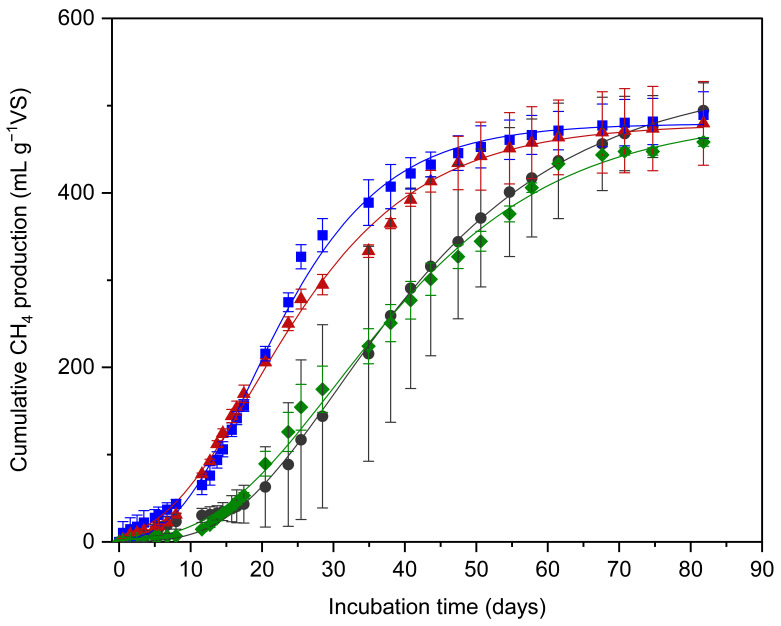
Cumulative methane production of mixed feedstocks subjected to various pretreatment methods over a 90-day digestion period. The symbols represent the experimental data (Symbols: ●: Untreated; ■: BM; ▲: BM + water; ◆: THP + BM), while the lines denote the predicted values estimated by the modified Gompertz model (black: Untreated; blue: BM; red: BM + water; green: THP + BM). Error bars indicate the standard deviation of triplicate measurements.

To evaluate the influence of physical and chemical changes induced by pretreatments on anaerobic biodegradability, the cumulative methane yield was measured through BMP tests, and the digestion kinetics were further analyzed. Figure 3 presents the cumulative methane production curves for each substrate.

Three distinct features were observed from the BMP results. First, the initial methane production rate varied depending on the pretreatment conditions. The cumulative methane production curves showed a steeper initial slope for the ball-milled treatments (BM and BM + water), indicating accelerated initial methanogenesis compared with the untreated and THP + BM conditions (Figure 3). This suggests that while all pretreatments enhanced solubilization, the specific chemical transformations in the ball-milled samples were more conducive to rapid initial microbial uptake. Second, despite the variations in initial rates, the experimental maximum methane production (*M*_exp_) measured at the end of the test showed no statistically significant differences among the treatments (*p* = 0.552). Although the untreated and THP + BM groups initially showed a slower increase, their cumulative productions eventually converged with those of the ball-milled groups toward the end of the digestion period. The *M*_exp_ values were 494.3, 489.5, 479.7, and 458.5 mL CH_4_ g^−1^ VS for the untreated, BM, BM + water, and THP + BM treatments, respectively. This indicates that while pretreatments influenced the kinetics, they did not significantly alter the ultimate biodegradability of the mixed substrate. Notably, while methane production from lignocellulosic biomass is often reported to reach a plateau within 35–40 days [7,23], the high lipid and protein content in our mixed slaughterhouse and agricultural waste necessitated an extended incubation period. Since the hydrolysis of complex lipids is often a rate-limiting step, monitoring was continued until a definitive production plateau was achieved for all treatments [24]. This approach ensured a reliable comparison of the ultimate methane potential without underestimating the yields from substrates with slower kinetics, such as the untreated and THP + BM groups.

Third, although *M*_exp_ remained similar, the variability (standard deviation) among replicates differed markedly across treatments. In particular, the THP + BM treatment exhibited a significantly lower standard deviation compared to the other conditions. This enhanced consistency in methane production is likely associated with the superior physical homogenization achieved through combined thermal hydrolysis and ball milling, which aligns with the lower SPAN value observed for the THP + BM condition (Section 3.1.2). Conversely, the untreated substrate showed remarkably high variability, reflecting the inherent heterogeneity of the mixed substrate. Notably, the standard deviation in the untreated and ball-milled groups during the log phase (Figure 3) frequently exceeded 5%. This is primarily attributed to the inherent heterogeneity of the mixed substrate, where even minor variations in the local concentration of lipids and proteins within each replicate can lead to divergent initial degradation rates. When such non-uniform substrates are applied to anaerobic digestion, they can lead to inconsistent reactor performance and significant fluctuations in gas production. Ultimately, achieving such physical uniformity and reduced variability through effective pretreatment is a crucial factor for the stable operation of anaerobic digestion systems, as it ensures predictable performance and process reliability regardless of the absolute methane yield.

The cumulative methane production data were further analyzed using the modified Gompertz model to quantify the kinetic behavior of each treatment. The model showed an excellent fit to the experimental results with high correlation coefficients (*R*^2^ > 0.99), and the derived kinetic parameters are summarized in Table 2. A detailed analysis of these kinetic parameters and their implications for process efficiency is provided in the following section.

### 3.3. Methane Production Kinetics and Lag Phase Analysis

The kinetics of methane production were quantitatively evaluated using the modified Gompertz model, with the resulting parameters summarized in Table 2. The high correlation coefficients (*R*^2^ > 0.99) across all treatments indicate that the model successfully captured the transition from the lag phase to active methanogenesis.

The most prominent effect of ball-milling was the significant reduction in the lag phase duration (λ). As shown in Table 2, the λ for the untreated substrate was 18.1 days, whereas the BM and BM + water treatments shortened this period to 7.7 and 6.1 days, respectively. This approximately threefold reduction in the lag phase demonstrates that ball-milling effectively enhanced substrate accessibility for methanogens, initiating the digestion process much earlier than the raw substrate. Consequently, the maximum methane production rate (*R*_m_) was highest in the BM treatment (17.1 mL g^−1^VS·d^−1^), further confirming the kinetic superiority of mechanical pretreatment.

**Figure 4:**
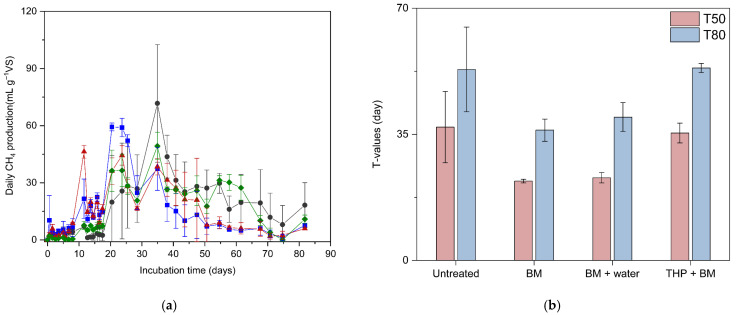
Daily methane production rates and kinetic time-point indicators. (**a**) Daily methane production profiles over 90 days of anaerobic digestion for untreated and pretreated mixed substrates (Symbols: ●: Untreated; ■: BM; ▲: BM + water; ◆: THP + BM). (**b**) *T*-values (*T*_50_ and *T*_80_) representing the time required to reach 50% and 80% of the ultimate methane yield, respectively.

These model-derived parameters are closely aligned with the daily methane production profiles presented in Figure 4a. The BM + water treatment reached its peak production rate (46.3 mL g^−1^VS) at 11.6 days, marking the earliest peak among all conditions. At this point, the untreated and THP + BM treatments exhibited substantially lower values (7.0 and 7.6 mL g^−1^VS, respectively), as they were still within their respective lag phases. Although the BM treatment reached its maximum daily production later (20.5 days), its peak value (59.3 mL g^−1^VS) was the highest recorded, reflecting the rapid conversion of accessible organic matter. The *T*-values further validate these kinetic trends (Figure 4b). The *T*_50_ values, representing the time required to reach 50% of the ultimate yield, were significantly reduced from 40.0 days (untreated) to 22.0 days (BM) and 22.9 days (BM + water) (*p* < 0.05). This indicates that ball-milling nearly halved the time required for major methane generation. Interestingly, the *T*_80_ values showed no statistically significant differences among the treatments (except for BM vs. THP + BM), suggesting that while the initial kinetics were dramatically improved, the cumulative production eventually converged as the easily degradable fractions were exhausted.

Overall, the results confirm that ball-milling-based pretreatments (BM and BM + water) are highly effective in advancing the initial stage of methane generation and shortening the overall digestion time. Although the untreated substrate showed a numerically higher maximum methane production potential (*P*_m_) of 534.4 mL g^−1^VS in the model (Table 2), this difference was not statistically significant (*p* > 0.05) as the cumulative production curves eventually converged at the later stage. However, it is noteworthy that the daily production rates (Figure 4a) exhibited high variability, with standard deviations exceeding 5% during the log phases of the untreated group. This phenomenon reflects the inconsistent timing of microbial colonization on the varied surfaces of the heterogeneous mixed waste, further emphasizing the inherent instability of digestion kinetics in untreated substrates. In this context, the primary benefit of the pretreatments was not an increase in the ultimate methane yield, but rather the dramatic acceleration of production kinetics, as evidenced by the significantly shorter *λ* and *T*_50_ values. This enhanced kinetics offer significant practical advantages for increasing the throughput and volumetric efficiency of anaerobic digesters. Additionally, while the THP + BM treatment exhibited a longer *λ* (13.3 days) than ball-milling alone, it showed much lower variability compared to the untreated control. This suggests that the combined thermal and mechanical energy effectively improved substrate homogenization, leading to more predictable and stable methane production profiles.

### 3.4. Temporal Profiles of VFAs

VFAs are key intermediates governing methane production pathways and reaction kinetics during anaerobic digestion. In particular, acetate serves as a direct substrate for acetoclastic methanogens, whereas excessive accumulation of VFAs can induce a decline in pH, leading to inhibition of methane production. Therefore, the temporal profiles of VFA concentrations and pH during BMP assays are analyzed in Section 3.4 and Section 3.5 to elucidate the delayed methane production observed for the untreated and THP + BM treatments in Section 3.3.

**Figure 5:**
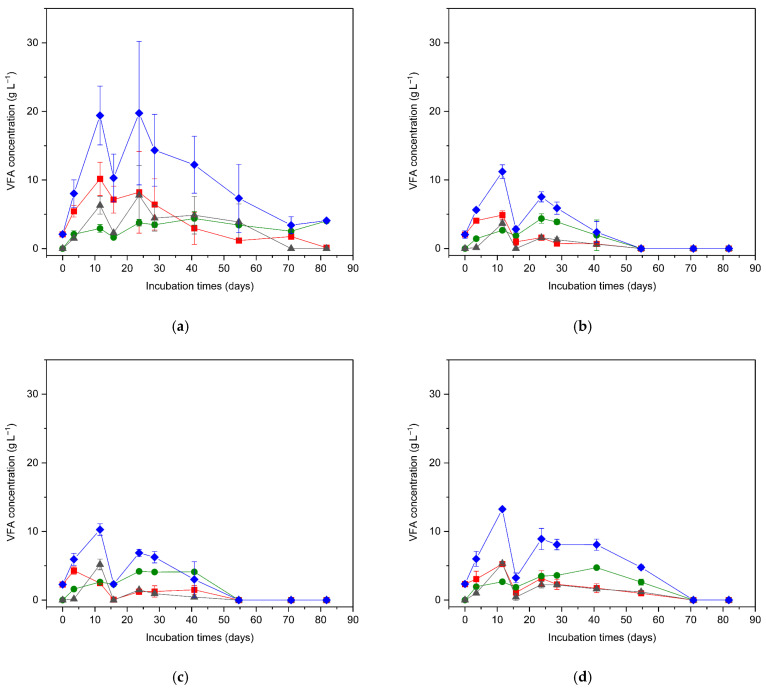
Temporal profiles of volatile fatty acid (VFA) concentrations during BMP assays: (**a**): untreated, (**b**): BM, (**c**): BM + water, and (**d**): THP + BM. Symbols indicate individual VFA species (■: acetate; ●: propionate; ▲: others), and ◆ represents the total VFA (TVFA) concentration. “Others” denotes the sum of all VFA species (butyric acid, iso-butyric acid, valeric acid, iso-valeric acid) excluding acetate and propionate.

In the untreated substrate, the total VFA (TVFA) concentration surged to 19.4 g L^−1^ by day 11.6, driven by rapid acetate accumulation (10.2 g L^−1^) and longer-chain VFAs (Figure 5a). Notably, while other VFAs were eventually consumed, propionate remained persistently high (3–4 g L^−1^) even after 40 days. This indicates a severe metabolic imbalance where hydrolysis and acidogenesis initially proceeded, but propionate oxidation—a thermodynamically demanding process—could not keep pace. This persistent VFA accumulation serves as a primary cause for the inhibited methane production in the raw substrate.

In contrast, the BM treatment effectively alleviated TVFA accumulation, maintaining a lower maximum concentration of 11.2 g L^−1^ (Figure 5b). Acetate was rapidly depleted by day 15.8, and all VFA species were fully consumed by day 54.6. This suggests that the reduced particle size and increased bioavailability induced by ball-milling allowed methanogens to synergize more effectively with acidogens. For BM + water, a transient accumulation of propionate was observed after day 20 (Figure 5c). This implies that the rapid degradation of other organic fractions temporarily overwhelmed the propionate-oxidizing syntrophs, creating a brief kinetic bottleneck.

The THP + BM treatment demonstrated higher initial VFA accumulation (13.3 g L^−1^) than the BM-only groups (Figure 5d). This was likely attributed to the enhanced solubilization of carbohydrates and proteins (Figure 2), which triggered a rapid burst of acidogenic activity. Although VFAs were eventually depleted by day 70.8, the slower consumption rate compared to BM suggests that the thermal pretreatment imposed a metabolic load that required a longer adaptation period for the microbial community.

The common observation of propionate accumulation across most experimental conditions highlights the stringent thermodynamic constraints for its degradation. Unlike acetate, propionate oxidation is thermodynamically favorable only under extremely low hydrogen partial pressures in the liquid phase, which necessitates a high level of syntrophic activity between acidogens and hydrogenotrophic methanogens [25]. Consequently, in systems where this syntrophic relationship is not yet fully established or is disrupted by rapid initial acidification—as observed in our experimental groups—propionate tends to persist as a kinetic bottleneck. Our results demonstrate that while ball-milling-based pretreatments promoted rapid acidogenesis, the overall process efficiency was ultimately determined by how effectively the microbial community transitioned through this propionate-degradation stage.

### 3.5. pH Fluctuations and Acid Stress

**Figure 6:**
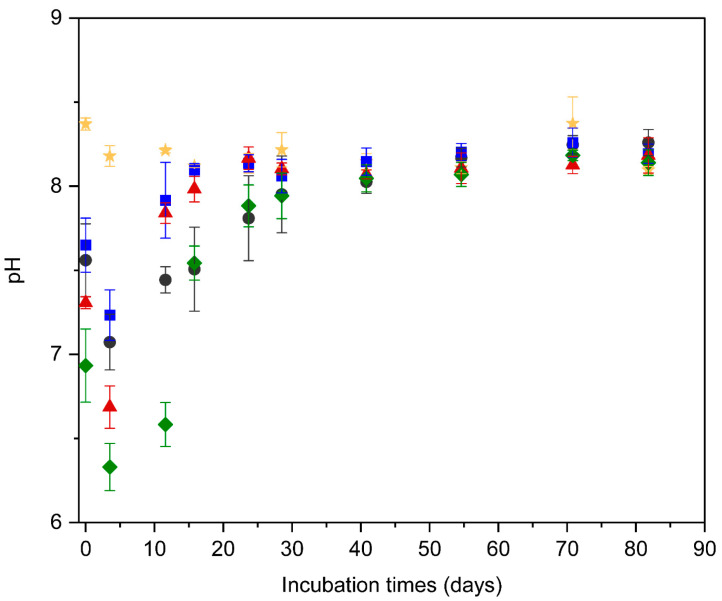
pH variation in digests during the BMP tests. pH values were measured at selected sampling days during the digestion period. Each panel shows for ★: sludge; ●: Untreated; ■: BM; ▲: BM + water; ◆: THP + BM.

The observed VFA dynamics were closely mirrored by variations in reactor pH, a critical parameter governing methanogenic activity (Figure 6). Monitoring the recovery of pH provides critical insights into the duration of inhibitory conditions and the timing of functional restoration in methane production. Throughout the digestion period, the inoculum sludge maintained a stable pH range of 8.0–8.4, serving as a baseline for the system. While initial pH drops were observed across all treatments following substrate addition, the recovery kinetics varied by pretreatment.

In the untreated substrate, the pH decreased to 7.1 at day 3.5 and exhibited a relatively slow recovery, remaining within the range of 7.0–8.0 for nearly four weeks before finally reaching 8.0 at day 40.8. In contrast, the BM treatment showed a much more resilient profile: although the pH dropped to 7.2, it rapidly recovered to 7.9 by day 11.6 and stabilized around 8.0 shortly thereafter. The BM + water treatment followed a similar pattern to the BM treatment; while it reached a lower pH of 6.7 at day 3.5, a sharp increase to 7.8 was observed by day 11.6, with the pH subsequently stabilizing within the range of 8.0–8.2 after day 15.8. The THP + BM treatment, however, exhibited the most severe acidification, with the pH plunging to a minimum of 6.3 at day 3.5. Similar to the Untreated group, the pH recovery in THP + BM was markedly delayed, remaining below the inhibitory threshold of 6.6—as identified by Sun (2020) [26]—during the critical initial phase.

As noted, the initial pH of the THP + BM treatment fell well within the inhibitory range of 6.6 identified by Sun (2020) [26]. More importantly, previous studies have demonstrated that the recovery of methanogenesis is highly dependent on the intensity and duration of initial acid stress [27,28]. Even after the pH returns to the optimal range (pH 7.0–8.0), sustained exposure to low pH can cause lasting physiological stress to methanogens, thereby delaying their functional restoration. This provides a plausible explanation for why THP + BM did not exhibit a rapid surge in methane production immediately after pH normalization, in stark contrast to the BM and BM + water treatments.

In summary, the initial methane production delays observed in the untreated substrate and the THP + BM treatment stemmed from distinct mechanisms. For the untreated substrate, limited physical accessibility hindered the hydrolysis stage, disrupting the metabolic balance and leading to VFA accumulation. Conversely, for the THP + BM treatment, while pretreatment effectively enhanced solubilization, it induced severe initial acid stress that delayed the recovery of the methanogenesis stage. These findings highlight that the choice of pretreatment dictates specific kinetic constraints on the anaerobic digestion process.

### 3.6. Process Implications: Pretreatment Severity and Comparative Assessment

The results of this study suggest that, for feedstocks with high organic content, such as slaughterhouse by-product, increasing the severity of pretreatment does not always translate to improved digestion efficiency. Due to the distinct mechanical and thermal mechanisms of the pretreatments used, “pretreatment intensity” was functionally defined in this study based on the metabolic response of the anaerobic system. Specifically, “excessive solubilization” was quantitatively indicated by the rapid initial rate of pH decline and volatile fatty acid (VFA) accumulation within the first 3.5 days. The rapid acidification and subsequent methanogenic delay observed in the THP + BM treatment indicate that excessive solubilization can overwhelm the microbial buffering capacity, particularly in protein-rich substrates where rapid ammonification and VFA production occur simultaneously. Consequently, the “threshold severity” for this high-strength substrate was identified as the level of treatment that triggers a pH decline below the inhibitory threshold of 6.6. While the ball-milled treatments remained above this threshold, the THP + BM treatment (minimum pH 6.3) clearly exceeded it, causing lasting physiological stress. This implies that rather than simply maximizing solubilization, it is crucial to establish an optimal ‘pretreatment severity’ tailored to the specific characteristics of the feedstock.

To evaluate this efficiency in a broader context, the methane yields achieved in this study (459–490 mL CH_4_ g^−1^VS) were compared with various technologies in the literature (Table 3). Although some studies using thermos-alkaline pretreatment (550 mL CH_4_ g^−1^VS) or hydrothermal methods (510 mL CH_4_ g^−1^VS) reported higher yields, these results were often obtained from highly biodegradable substrates, such as pre-processed or ‘cooked’ food waste, which inherently easier to degrade. In contrast, our study utilized a mixture of raw agricultural residues and complex slaughterhouse by-products, characterized by a highly recalcitrant lipid-protein matrix. Despite the challenging nature of these substrates, our ball-milling-based approach achieved highly competitive yields without the requirement for chemical additives, demonstrating its robust potential for handling diverse and physically complex waste streams.

For unutilized biomass with high organic loading, a balanced approach is required to enhance physical accessibility without inducing excessive initial metabolic stress. Future research should focus on optimizing the intensity of thermal-mechanical pretreatments to identify a “threshold severity” that prevents methanogenic inhibition. Furthermore, integrating active pH-control strategies, particularly in continuous reactor systems, could be a viable path to mitigate acid stress and fully harness the benefits of enhanced solubilization. While current biochemical methane potential results indicate this necessity, verification in continuous systems is required for practical application.

Looking forward, the sustainable valorization of complex mixed feedstocks—such as the slaughterhouse and agricultural residues explored here—requires a multi-faceted approach. Future research should prioritize the optimization of co-digestion ratios to achieve an ideal C/N balance, mitigating the risk of ammonia inhibition inherent in nitrogen-rich animal wastes. Furthermore, to overcome the kinetic bottlenecks and acid stress observed in high-severity pretreatments, the integration of conductive catalysts (e.g., biochar, magnetite, or carbon nanotubes) presents a promising direction [31,32,33]. These materials can facilitate direct interspecies electron transfer (DIET) between syntrophic bacteria and methanogens, thereby accelerating VFA consumption and enhancing methane production stability. Combining tailored pretreatment intensity with such catalytic strategies will be crucial for scaling up the anaerobic digestion of high-strength organic waste into stable, high-efficiency industrial systems.

## 4. Conclusions

This study evaluates the effects of ball-milling and combined thermal hydrolysis and ball-milling pretreatments on the anaerobic digestion performance and reaction kinetics of a mixed substrate of slaughterhouse by-products and agricultural residues. The results demonstrate that while ball-milling-based pretreatments did not significantly alter the ultimate methane yield, they markedly improved reaction kinetics by reducing the lag phase and increasing the maximum methane production rate.

Specifically, the ball-milling treatment effectively stabilized the initial digestion stage and accelerated the onset of methanogenesis, proving to be an efficient mechanical strategy for enhancing substrate accessibility. In contrast, the combined thermal hydrolysis and ball-milling treatment exhibited limited kinetic enhancement despite achieving superior substrate solubilization. This was attributed to a severe metabolic imbalance characterized by rapid volatile fatty acid accumulation and initial pH drops below the inhibitory threshold (6.6), which imposed lasting physiological stress on methanogens.

The findings suggest that, for high-strength organic biomass, the “severity” of pretreatment must be strategically optimized; excessive solubilization can overwhelm the microbial buffering capacity and delay functional recovery. To address these kinetic constraints, future research should evaluate active pH-control strategies within continuous reactor systems. While the current biochemical methane potential results highlight the necessity of such interventions to mitigate initial acid stress, verification in continuous configurations remains essential for successful large-scale applications.

## Figures and Tables

**Table 1:** Physicochemical characteristics of slaughterhouse by-products and agricultural residues used in this study.

Biomass		W·C ^(1)^ (%)	TS ^(2)^ (mg/kg)	VS ^(3)^ (mg/kg)	VS/TS (%)	TKN ^(4)^ (mg/kg)	TKN/VS (%)
Agricultural residues		90.6	93,769	85,814	91.5	-	-
Slaughterhouse by-product	Residual cake	56.7	433,424	430,702	99.4	10,579	2.5
	Liver	70.8	291,636	273,339	93.7	29,632	10.8
	Skin waste	71.8	282,727	256,116	92.7	27,624	10.8
	Contents in stomach	75.3	247,199	225,969	91.3	4508	2.0
	lung	80.9	191,301	175,439	91.7	23,782	13.6

(1) Water content; (2) Total solid; (3) Volatile solid; (4) Total kjeldahl nitrogen.

**Table 2:** Kinetic parameters of the modified Gompertz model for methane production form untreated and pretreated substrate.

	Untreated	BM	BM + Water	THP + BM
Maximum methane production potential (*P*_m_) (mL g^−1^VS)	534.4 ± 49.5	479.4 ± 21.2	479.3 ± 53.6	487.5 ± 18.4
Lag phase duration (*λ*) (d)	18.1 ± 5.7	7.7 ± 0.8	6.1 ± 0.5	13.3 ± 1.1
Maximum methane production rate (*R*_m_) (mL g^−1^VS·d^−1^)	13.0 ± 2.9	17.1 ± 1.6	14.0 ± 0.8	10.6 ± 0.6
Correlation coefficient (*R*^2^)	>0.99	>0.99	>0.99	>0.99

**Table 3:** Comparative assessment of methane yields from various pretreatment methods and applied to single and mixed substrates.

Pretreatment Methods	Digestion Type	Substrate	Methane Yield (mL g^−1^VS)	Reference
Thermo-alkaline	Co-digestion	Slaughterhouse + food waste *	550	[29]
Hydrothermal	Single	Poultry slaughterhouse wastes	480	[16]
Microwave	Single	Slaughterhouse sludge	490	[30]
Ball-milling	Co-digestion	Slaughterhouse waste + agricultural residue	490	This study
Combined THP and BM	Co-digestion	Slaughterhouse waste + agricultural residue	459	This study
Ball-milling	Single	Food waste *	510	[18]

* Food waste is considered a highly biodegradable substrate due to prior processing (e.g., cooking).

## Data Availability

The data presented in this study are available on request from the corresponding author.

## Supplementary Materials

The following supporting information can be downloaded at https://www.mdpi.com/article/10.3390/bioengineering13030326/s1. Table S1: Cumulative particle size distribution parameters (D_10_, D_50_, and D_90_) of the mixed substrate under different pretreatment methods (μm). Table S2: Physicochemical properties of untreated and pretreated substrates (%).

## Abbreviations

The following abbreviations are used in this manuscript:

BM	Ball-milling
BM + water	Ball-milling with water
THP + BM	Combined thermal hydrolysis and ball-milling
BMP	Biochemical Methane Potential
IPCC	Intergovernmental Panel on Climate Change
AR6	Sixth Assessment Report
SLCF	Short-Lived Climate Forces
AD	Anaerobic Digestion
FAOSTAT	Food and Agricultural Organization Corporate Statistical Database
LCFA	Long Chain Fatty Acid
TKN	Total Kjeldahl Nitrogen
TS	Total Solid
VS	Volatile Solid
APHA	American Public Health Association
S-TOC	Soluble Total Organic Carbon
BCA	Bicinchoninic Acid
VFA	Volatile Fatty Acid
ANOVA	Analysis of Variance
HSD	Honestly Significant Difference
M_exp_	Maximum Methane Production
λ	Lag Phase Duration
Rm	Maximum Methane Production Rate
Pm	Maximum Methane Production Potential
