# Supplementary material for: Impact of Ball-Milling and Thermal Hydrolysis on Physicochemical Properties and Anaerobic Digestion Kinetics of Mixed Slaughterhouse and Agricultural Wastes
Source: Bioengineering (Basel). 2026 Mar 11;13(3):326. doi: 10.3390/bioengineering13030326 (PMC13024333; doi:10.3390/bioengineering13030326)
Supplement: Supplementary file 1 [file bioengineering-13-00326-s001.zip › bioengineering-4189710-supplementary.pdf]

Impact of Ball-Milling and Thermal hydrolysis on Physicochemical Properties and Anaerobic Digestion Kinetics of Mixed Slaughterhouse and Agricultural Wastes

Sang Heon Lee[1,2], Oh hyun Gweon[1], Hye Sun Lee[1], Byoung Seung Jeon[1], Youngwook Go[2], Chang Sook Jin[3], Youngseop Yu[3], Byoung-In Sang[2], and Jin Hyung Lee[1,*]

[1] Korea Institute of Ceramic Engineering and Technology, Cheongju 28160, Republic of Korea
[2] Department of Chemical Engineering, Hanyang University, Seoul 04763, Republic of Korea
[3] KEC system Co., Hwasung 18244, Republic of Korea
* Correspondence: leejinh1@kicet.re.kr; Tel.: +82-43-913-1502

Table S1. Cumulative particle size distribution parameters (D_{10}, D_{50}, and D_{90}) of the mixed substrate under different pretreatment methods (μm).

	BM	BM+water	THP+BM
**D_{10}**	6.53 ± 0.31	8.13 ± 0.40	6.71 ±0.39
**D_{50}**	75.17 ± 16.91	110.94 ± 37.73	66.80 ± 2.42
**D_{90}**	674.04 ± 191.28	550.69 ± 146.04	239.80 ± 10.39

Note: D_{10}, D_{50}, and D_{90} represent the particle diameters at 10%, 50%, and 90% of the cumulative volume, respectively.

Table S2 Physicochemical properties of untreated and pretreated substrates (%).

	untreated	BM	BM+water	THP+BM
Water contents	84.53 ± 0.38	85.72 ± 1.57	85.49 ± 1.53	83.54 ± 0.54
TS	15.47 ± 0.38	14.28 ± 1.57	14.51 ± 1.53	16.46 ± 0.54
VS	13.63 ± 0.46	12.14 ± 1.55	12.77 ± 1.13	14.79 ± 0.63

Note: TS: total solids, VS: volatile solids.
